# Supplementary material for: A general model of focal adhesion orientation dynamics in response to static and cyclic stretch
Source: Commun Biol. 2018 Jun 28;1:81. doi: 10.1038/s42003-018-0084-9 (PMC6123675; doi:10.1038/s42003-018-0084-9)
Supplement: Supplementary file 1 — Supplementary Information [file 42003_2018_84_MOESM1_ESM.pdf]

Supplementary Information

Supplementary Figure 1

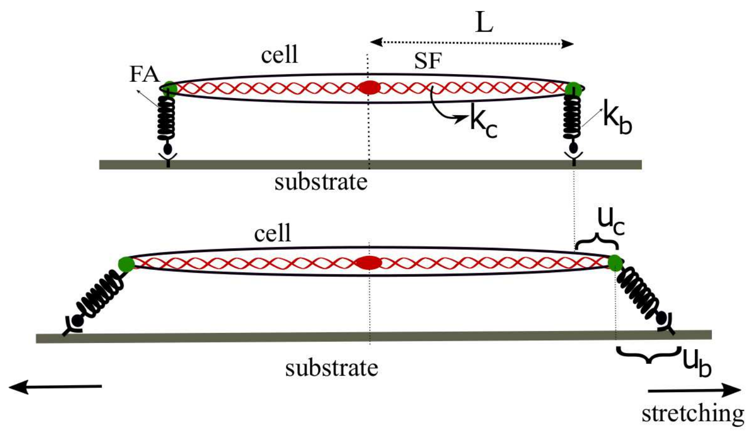

Supplementary Figure 1. Schematic diagram of the cell and the stress fiber (SF) adhering through two focal adhesions (FAs) under substrate stretching. The ligand-receptor bond is modelled as Hookian spring of spring constant k_b and the elasticity of the cell/stress fiber is represented by a spring of rigidity k_c. The bond displacement along the stretch direction is denoted by u_b and the cell spring displacement by u_c.
